# WHAT’S OLD IS NEW: USING ARTIFICIAL INTELLIGENCE TO ACCELERATE DISCOVERY OF NEW TREATMENTS

**DOI:** 10.1093/geroni/igz038.060

**Published:** 2019-11-08

**Authors:** Connie Marras, Laura C Maclagan, Yi Cheng, Naomi Visanji, Mina Tadrous, Susan E Bronskill

**Affiliations:** 1 Krembil Research Institute, University Health Network, Toronto, Ontario, Canada; 2 ICES, Toronto, Ontario, Canada; 3 Toronto Western Hospital, University Health Network, Toronto, Ontario, Canada; 4 St Michael’s Hospital, Toronto, Ontario, Canada

## Abstract

Given the high cost of drug development and low success rates, repurposing drugs already proven safe provides a promising avenue for identifying effective therapies with additional indications. The IBM Watson artificial intelligence system was used to search 1.3 million Medline abstracts to prioritize medications that may be potentially disease-modifying in Parkinson’s disease. We assessed patterns of use of the top 50 Watson-ranked drugs among 14,866 adults with Parkinson’s disease aged 70 and older who were matched to persons without Parkinson’s disease on age, sex, and comorbidity. Sociodemographic characteristics, chronic conditions, and use of other medications were compared using standardized differences. Patterns of potentially disease-modifying drug use were examined prior to and following ascertainment of Parkinson’s disease. Preliminary findings from multivariable conditional logistic regression models on the association between previous exposure to potentially disease-modifying drugs and Parkinson’s disease diagnosis will be presented.

